# Dentinal tubule occlusion using Er:YAG Laser: an *in vitro* study

**DOI:** 10.1590/1678-7757-2020-0266

**Published:** 2021-03-25

**Authors:** Hongmin ZHUANG, Yuee LIANG, Shaowen XIANG, Huanying LI, Xingzhu DAI, Wanghong ZHAO

**Affiliations:** 1 Southern Medical University Nanfang Hospital Department of Stomatology Guangzhou Guangdong China Southern Medical University, Nanfang Hospital, Department of Stomatology, Guangzhou, Guangdong, China.; 2 Guangzhou Hospital of Integrated Traditional and West Medicine Department of Stomatology Guangzhou China Guangzhou Hospital of Integrated Traditional and West Medicine, Department of Stomatology, Guangzhou, China.; 3 Southern Medical University Stomatological Hospital Guangzhou Guangdong China Southern Medical University, Stomatological Hospital, Guangzhou, Guangdong, China.

**Keywords:** Er:YAG laser, Dentin hypersensitivity, Power, Scanning electron microscopy, Temperature

## Abstract

**Objectives:**

We analyzed the effects of the Er:YAG laser used with different parameters on dentinal tubule (DT) occlusion, intrapulpal temperature and pulp tissue morphology in order to determine the optimal parameters for treating dentin hypersensitivity.

**Methodology:**

Dentin specimens prepared from 36 extracted human third molars were randomized into six groups according to the treatment method (n=6 each): control (A); Gluma desensitizer (B); and Er:YAG laser treatment at 0.5 W , 167 J/cm2 (50 mJ, 10 Hz) (C), 1 W , 334 J/cm2 (50 mJ, 20 Hz) (D), 2 W , 668 J/cm2 (100 mJ, 20 Hz) (E), and 4 W and 1336 J/cm2 (200 mJ, 20 Hz) (F). Treatment-induced morphological changes of the dentin surfaces were assessed using scanning electron microscopy (SEM) to find parameters showing optimal dentin tubule occluding efficacy. To further verify the safety of these parameters (0.5 W, 167 J/cm^2^), intrapulpal temperature changes were recorded during laser irradiation, and morphological alterations of the dental pulp tissue were observed with an upright microscope.

**Results:**

Er:YAG laser irradiation at 0.5 W (167 J/cm^2^) were found to be superior in DT occlusion, with an exposure rate significantly lower than those in the other groups (P<0.05). Intrapulpal temperature changes induced by Er:YAG laser irradiation at 0.5 W (167 J/cm2) with (G) and without (H) water and air cooling were demonstrated to be below the threshold. Also, no significant morphological alterations of the pulp and odontoblasts were observed after irradiation.

**Conclusion:**

Therefore, 0.5 W (167 J/cm^2^) is a suitable parameter for Er:YAG laser to occlude DTs, and it is safe to the pulp tissue.

## Introduction

Dentin hypersensitivity (DH) is one of the most frequently encountered chronic conditions characterized by transient and sharp tooth pain evoked by external stimuli, including thermal, evaporative, tactile, osmotic, and chemical stimuli. The discomfort caused by DH cannot be ascribed to any other dental defect or pathology.^1^According to Splieth and Tachou, et al.^2^ (2013) 3%–98% of individuals are affected by DH, which can cause varying degrees of irritation during eating, drinking, and even breathing.

Although the DH mechanism remains controversial, the theory of hydrodynamics is the most accepted. It suggests that external stimulation of teeth with DH results in fluid displacement within the dentinal tubules (DTs),^3^ which activates the nerve endings located at the pulp–dentin interface and eventually results in pain and discomfort. According to the theory of hydrodynamics, DT narrowing or occlusion for minimizing dentin permeability and lowering the pulp sensitivity threshold is a potential strategy for pain relief. Frequently used desensitizing agents can be classified into four categories: anti-inflammatory agents (corticosteroids), protein precipitants (formaldehyde, silver nitrate, strontium chloride hexahydrate), tubule-occluding agents (calcium hydroxide, potassium nitrate, sodium fluoride), and tubule sealants (resins and adhesives).^4^However, none of these agents can produce long-lasting effects, since abrasion and erosion by internal and external acids would lead to re-exposure of DTs over time.^5^

The advent of laser treatment has provided an alternative modality for DH management.^6^ Currently used lasers for this purpose include Nd:YAG lasers, Er:YAG lasers, Er,Cr:YSGG lasers, carbon dioxide lasers, and diode lasers.^7,9^ Among these, Er:YAG lasers with a wavelength at 2940 nm exhibit high absorption in water and are expected to minimize thermal damage to the pulp and dentin tissues.^10^ Walsh and Cummings^11^ (1994) found that water absorption was 15 and 10,000 times greater with Er:YAG lasers than with CO_2_ and Nd:YAG lasers, respectively. Therefore, due to the high water absorption peak compared to other commercially available lasers, Er:YAG lasers have gained popularity in clinical settings for treating oral diseases after it was approved by the U.S. Food and Drug Administration in 1997.^12^ When it comes to clinical practice in treating DH, parameters vary between brands due to differences in setups (Table 1). However, few studies have evaluated the optimal parameters for the Er:YAG laser in terms of DH treatment.^13-14^

**Table 1:** Different Parameters of Er:YAG Laser of Different Brands Treating Dentin Hypersensitivity

Brands	Power (W)	Frequency (Hz)	Energy/Pulse(mJ)	Irridiation Time(s)	Irridiation Area(mm^**2**^)	Distance (mm)	Applicator device	Water spray
Erwin	-	10	40	47	4	Slight contact	-	yes
DE- Light	-	30	60	10	-	3~4	Straight quartz round tip	no
Smart 2940 d plus, Deka	-	10	100	60	-	1	R14 handpiece	yes
Fidelis III,Fotona	0,2	3	80	120	7,74	6	R02-handpiece	yes
Fidelis Plus,Fotona	-	30	100	60s/cm2	-	1~2	R07-handpiece	yes
Key laser 3+, KaVo	-	2	40	40	4	25	2060 handpiece	no
Key Laser 1243,KaVo	-	2	60	Four irradiation of 20s, with a 1-min interval	9	6	2055 handpiece	No, only air cooling

In this study, we assumed the Er:YAG laser with optimal parameters can effectively treat DH by occluding DT and had no damage to the dental pulp. Therefore, the objective of this *in vitro* study is to explore the parameters of the Er:YAG laser when used for dentinal tubule occlusion to provide guidance for the clinical treatment of DH. As such, we investigated the effects of laser irradiation using these parameters on intrapulpal temperature changes and the morphological alterations in odontoblasts and pulp tissue were observed to determine the safety of Er:YAG laser in the treatment of DH.

## Methodology

### Study design

**Figure 1:**
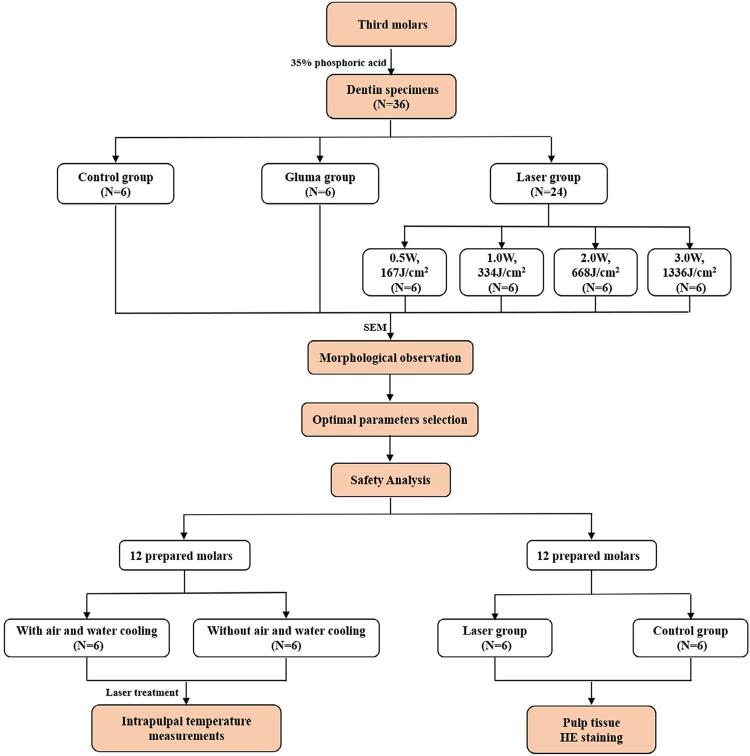
Flow diagram of the study

An *in vitro* study was conducted and the study protocol (Figure 1) was reviewed and approved by the hospital’s Institutional Review Board with a reference number NFEC-201701-K1-01.

### Preparation of dentin specimens

Human third molars extracted from adults aged 20–25 years old were thoroughly cleaned and inspected under magnification (×20). Those with cracks, caries, and restorations were discarded. Eventually, 36 molars were selected. Dentin specimens (DSs) with 2 mm thickness and 3×3 mm^2^ area were prepared from all 36 teeth using a high-speed diamond bur (Mani Inc., Japan) under water irrigation. In a direction parallel to the occlusal surface, enamel was removed up to 2 mm below the central fossa so that dentin was exposed. For homogeneous dentin surfaces, 200-, 600-, and 800-grit silicon carbide papers (SUISUN Ltd., HK, China) were used for polishing the specimens, which were then washed with a large amount of distilled water and disinfected by storage in distilled water with 0.2% thymol (ZhiYuan Ltd., Tianjin, China) for no more than 1 week until further use. Before the experiment, all specimens were conditioned with 35% phosphoric acid for 1 min (3M ESPE, St Louis, MN, USA) for DT exposure.

### Er:YAG laser Treatment

Following dentin exposure, the teeth were divided into six groups of six teeth each (according to random number table). Group A (control group) received no further treatment after exposure to 35% phosphoric acid. In group B, Gluma desensitizer (GD; Heraeus, Germany) was gently applied using cotton pellets, and the treated specimens were set aside for 60 s. Then, they were dried until the dentin surfaces lost their shine and subsequently rinsed with distilled water. The same procedure was performed twice. The specimens in groups C–F received Lite Touch Er:YAG laser (Lite Touch, Syneron Medical Ltd., Israel) irradiation at a wavelength of 2490 nm under the following sets of parameters: group C, 0.5 W, 167 J/cm^2^ (50 mJ, 10 Hz); group D, 1 W, 334 J/cm^2^ (50 mJ, 20 Hz); group E, 2 W, 668 J/cm^2^ (100 mJ, 20 Hz); and group F, 4 W, 1336 J/cm^2^ (200 mJ, 20 Hz). The other conditions remained the same for all groups (Table 2). Laser energy was delivered via the Magnum tip (green O-rings; length: 6.3 mm, diameter: 1.3 mm), which was placed at a 1-cm distance, under a water spray at level 1 for 30 s. During irradiation, the tip was moved to a mesiodistal direction at a speed of approximately 1 mm/s, and the irradiation area of specimens was 3*3 mm^2^. All irradiation procedures were performed by a single researcher to ensure treatment of the entire dentin surface with minimum variations.

**Table 2:** Parameters of Laser Groups

Groups	N	Energy(mJ)	Frequency(Hz)	Power(W)	Energy density(J/cm^**2**^)
Group C	6	50	10	0,5	167
Group D	6	50	20	1	334
Group E	6	100	20	2	668
Group F	6	200	20	4	1336

### SEM observation

The treated specimens were fixed in 2.5% glutaraldehyde (Phygene Com., Fuzhou, China) for 24 h at room temperature, rinsed with 0.1 M phosphate-buffered saline for glutaraldehyde removal, and air-dried. Then, they were dehydrated in a series of alcohol solutions (ZhiYuan Ltd., Tianjin, China) (30%, 50%, 70%, 80%, 95%, 100%; 15 min for each), sputter-coated with a layer of gold, and observed under a scanning electron microscope (S-4800 SEM, Hitachi Ltd., Hitachinaka, Japan) at 1500× and 5000× magnification.

The area of open or partially obliterated DTs observed by scanning electron microscopy (SEM) was measured by software (Image-Pro PLUS 6.0, Media Cybernetics, USA). On the basis of pixel grey value differences, the software can differentiate these DTs by drawing their outlines, thus facilitating calculation of the area of open or partially obliterated DTs. The tubule exposure rate for each group was subsequently calculated using the following formula:


$$\frac{\text{(exposure rate = mean total area of open or partially obliterated DTs) }}{\text{ mean total area }}$$

### Intrapulpal temperature measurements

In the preceding experiments, the surface of dental specimens treated with parameters 0.5 W, 167 J/cm^2^ (50 mJ, 10 Hz) has shown the most desirable structural changes without microcracks and carbonization, so we chose these parameters for the follow-up experiments. Twelve freshly extracted third molars were prepared and irradiated with parameters of 0.5 W, 167 J/cm^2^ (50 mJ, 10 Hz).

**Figure 2:**
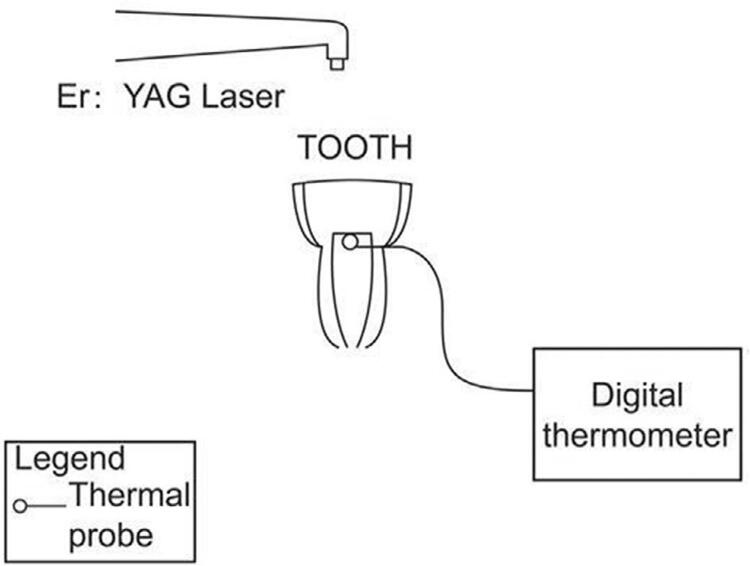
Schematic diagram of detection of temperature changes in pulp chamber

Before being irradiation, enamel was removed up to 2 mm in depth below the central fossa in a direction parallel to the occlusal surface so that dentin was just exposed. A diamond bur was used to mark an irradiation area of 3×3 mm^2^ at the center of the dentin. A hole with a diameter of 1 mm was created subjacent to the dentinoenamel junction to create access for the insertion of a type K thermocouple (diameter: 1 mm) into the pulp chamber. The type K thermocouple was connected to a digital thermometer (DT-610B, CEM, China). A thermal paste (TaoXin Com., Shenzhen, China) was introduced into the pulp chamber to ensure good contact between the tip of the thermocouple and the ceiling of the chamber (Figure 2). The heat conductivity of this paste was similar to that of the dental pulp. The root was sealed by glass ionomer cement (GC Fuji IX, Tokyo). After inserting the thermal paste and thermocouple, the cervical hole was sealed with wax.

Irradiation was performed with (group G; n=6) and without (group H; n=6) air and water cooling, while other conditions remained the same as those described earlier. Temperature changes during irradiation were recorded at 5 or 10 s intervals by calculating the difference between the recorded values and the initial temperature values.

### Morphological alterations of pulp tissue

Twelve healthy third human molars were selected to remove coronal enamel to just expose dentin beneath, yielding twelve dentin specimens. They were divided randomly into 2 groups, as laser group (group A, 0.5 W, 167 J/cm^2^) and control group (group B). Following our previous outcomes, the laser group was applied with a treatment using parameters of 0.5 W, 167 J/cm^2^, while the control group was treated with nothing. They were cut longitudinally to take the pulp tissue. HE (hematoxylin-eosin) staining was used to observe pulp histomorphology by light microscopy (Olympus BX51; Olympus Optical Co., Ltd., Tokyo, Japan).

### Statistical analysis

All collected data were statistically analyzed using SPSS version 23.0 (SPSS Inc., Chicago, Illinois, USA). Multiple intergroup comparisons were performed using the Kruskal–Wallis test. When this test presented a significant difference, the multiple (double) comparison Mann–Whitney U test was used. A p-value of <0.05 was considered statistically significant.

## Results

### SEM observation

**Figure 3:**
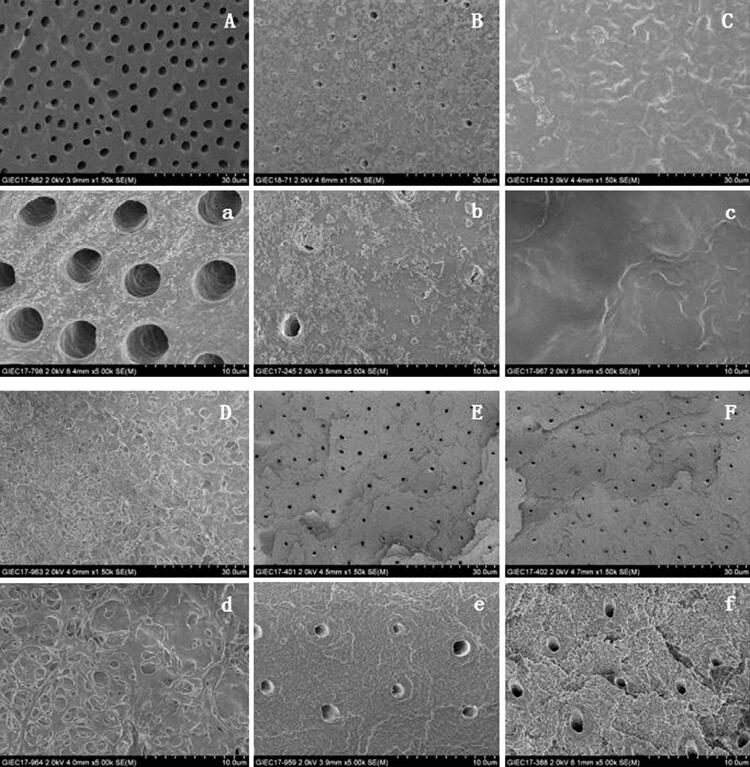
SEM Micrographs of treated dental specimens of group A (A,a; ×1500,×5000), group B (B,b; ×1500,×5000), group C (C,c; ×1500,×5000), group D (D,d; ×1500,×5000), group E (E,e; ×1500,×5000), group F (F,f; ×1500,×5000)

SEM images for the control group showed numerous exposed DTs parallel to each other, without plugged debris (Figure 3: A, a). The micrograph for the Gluma desensitizer group revealed the occlusion of several DTs by precipitant plugs, with a few partially occluded DTs (Figure 3: B, b). In group C (0.5 W, 167 J/cm^2^), a thick, smooth, melted layer covering the superficial dentin surface was observed (Figure 3: C, c). The DTs were almost completely obliterated by this layer. In group D (1 w, 334 J/cm^2^), the dentin surface appeared to be melting with the formation of bubbles, and a few partially occluded DTs were observed (Figure 3: D, d). The other two groups (groups E and F), which involved the use of stronger powers, revealed very similar, scale-like surfaces with open tubules of different depths (Figure 3: E, e, F, f).

**Figure 4:**
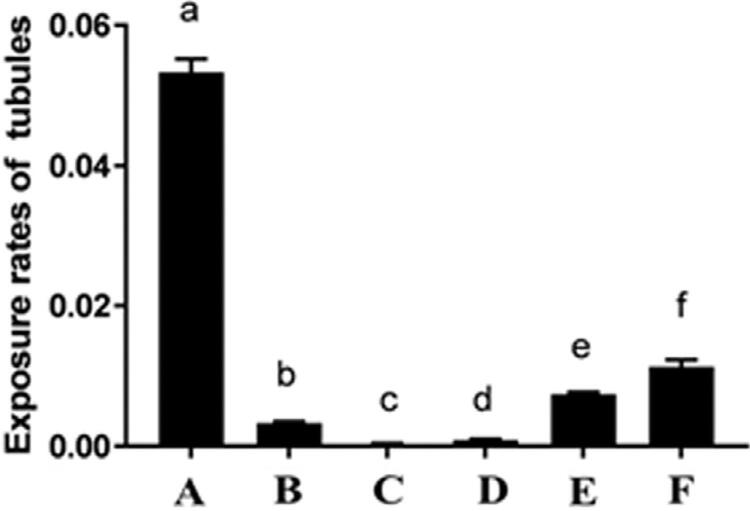
Comparisons of Exposure rates of tubules. a–f indicate statistically significant differences between groups (P<0.05)

Figure 4 shows comparisons of the exposure rates between groups. There were significant differences among all six groups (*P*<0.001). The tubule exposure rate of the Gluma desensitizer treatment group (group B) was significantly lower than that of the control group (*P*<0.05), but still higher than the exposure rates of groups C and D (*P*<0.05). In laser groups, the exposure rate in group C (0.0002±0.0002) was significantly lower than that of other groups (*P*<0.05), and the exposure rate evidently increased with an increase in power (*P*<0.05).

### Intrapulpal temperature measurements

**Figure 5:**
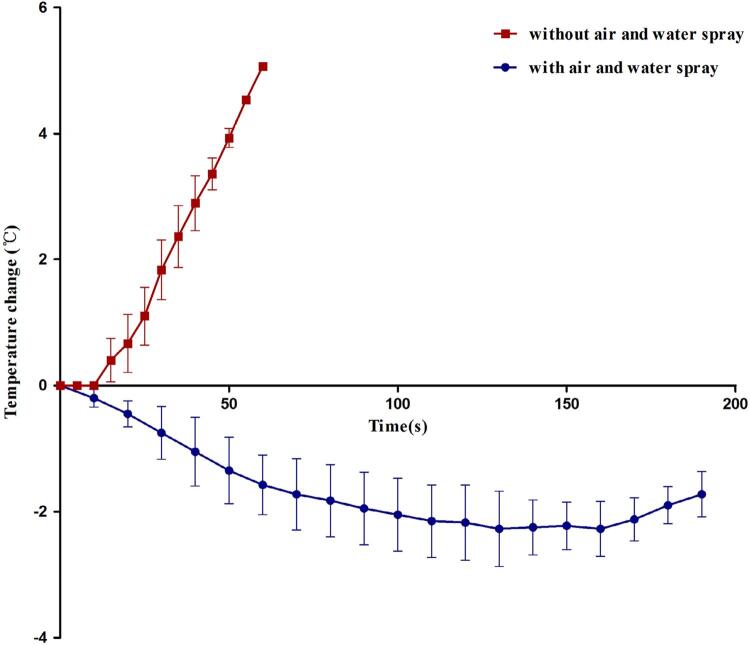
Intrapulpal temperature change during Er:YAG laser irradiation (0.5 W , 167 J/cm2) with and without air and water spray cooling

On the basis of the favorable results obtained for group C in the preceding experiments, we used the irradiation parameters of 0.5 W and 167 J/cm^2^ for intrapulpal temperature measurements. Figure 4 shows the results of the intrapulpal temperature measurements during Er:YAG laser irradiation at 0.5 W and 167 J/cm^2^. Under air and water cooling, the final temperatures were lower than the temperature registered before irradiation. The temperature gradually decreased by −2.275±0.597°C and gradually increased thereafter, with a change of −1.725°C ± 0.359°C recorded at 190 s. In contrast, laser irradiation without air and water cooling for 60 s resulted in a temperature change of 5.067°C±0.058°C (Figure 5).

Intrapulpal temperature change during Er:YAG laser irradiation (0.5 W , 167 J/cm^2^) with and without air and water spray cooling.

### Morphological alterations of pulp tissue

**Figure 6:**
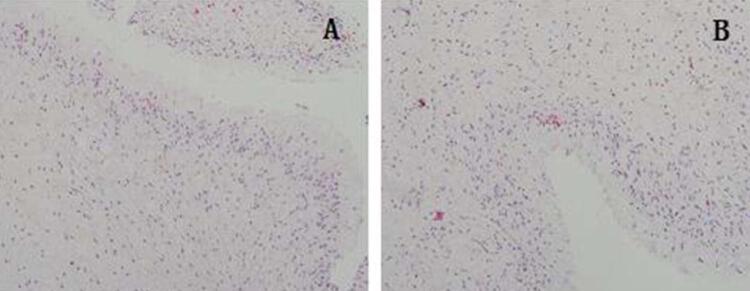
Micrograph of pulp tissue after HE staining ×200. (A: control group; B: laser group, 0.5 W , 167 J/cm2)

The morphology of the pulp was observed by light microscopy after HE staining. No significant differences were observed between the two groups. The morphology of the odontoblast cells and vessels, as well as of the collagenic and neural fibers, was clear and healthy (Figure 6).

## Discussion

In this *in vitro* study, Er:YAG laser with the parameters of 0.5 W, 167 J/cm^2^ (50 mJ, 10 Hz) under a water spray at level 1 was effective in occluding dentin tubules and harmless to the dental pulp, which provided the theoretical basis for the treatment of dentin hypersensitivity.

Absi, Addy and Adams^15^ (1987) showed a number of open DTs per surface area eight times greater in teeth with DH than in those without DH, and the tubular diameter was two times greater in sensitive teeth than in insensitive teeth. Moreover, there was a comparative study suggesting that 35% phosphoric acid resulted in better DT exposure than 24% ethylenediamine tetraacetic acid (EDTA) did when under SEM.^16^ Therefore, in the present study, DTs in DSs were exposed to 1-min application of 35% phosphoric acid to establish DH models. SEM images for our control group showed clean and smooth dentin surfaces with tubule orifices that were free of smear layers and plugs; these findings were consistent with those of previous studies.^10,12,14^

Gluma desensitizer is composed of glutaraldehyde and 2-hydroxyethyl-methacrylate (HEMA), which coagulates the serum albumin in dentinal fluid. This reaction between glutaraldehyde and albumin induces HEMA polymerization.^17,18^ Thus, the desensitizer can form a coagulation plug similar to the melted layer formed after laser irradiation. We used the gluma desensitizer as a positive control in the present study, in accordance with several other studies.^19,20^ There is lack of consensus over whether laser serves as a better option in treating DH than Gluma desensitizer. An 18-month randomized clinical study conducted by Lopes, Euardo e Aranha^21^ (2017) showed that compared to the Nd:YAG laser treatment group and the Nd:YAG laser+Gluma desensitizer treatment group, the Gluma desensitizer treatment group had the most prolonged duration on desensitizing. However, Ozlem, et al.^1^ (2018) used the yeaple probe to evaluate the dentin sensitivity of patients with dentin hypersensitivity treated by Er:Cr:YSGG laser or Gluma desensitizer or a combination of the two. The results showed that using Er:Cr:YSGG laser to treat the disease alone could get the most desirable results even at different time intervals (7, 90, 180 days).^1,21^ Considering that the wavelength of Er:YAG laser is closed to that of the Er:Cr:YSGG laser, the principle of action of the two lasers in occluding dentin tubules is similar. The excellent efficacy of Er:Cr:YSGG laser could serve as a solid foundation for the promising application prospects of Er:YAG laser in treating DH.

Er:YAG lasers are high-power lasers, and we used powers of 0.5 (lowest) to 4 W in the present study. According to Table 1, the Er:YAG laser parameter settings for desensitization treatment are usually low (the output power range is between 0.08 W-3 W) and, as for the application of the cooling system, when the output power is high (3 W), the laser irradiation should be accompanied by water, whereas when the output power is low (0.08 W), laser irradiation could work without water. Considering that the lowest built-in parameter of the laser used in this experiment is set to 0.5 W, we set 0.5W as the starting value for parameter exploration, and at the same time turned on the water-air mode for safety reasons. The laser-treated groups exhibited significant differences in SEM findings. Moreover, the DT exposure rate was the lowest after irradiation at 0.5 W and 167 J/cm^2^, with the specimens showing almost complete DT occlusion by the melted layer on SEM images. Our findings were consistent with the findings of previous studies exploring the DT occluding effects of the Er:YAG laser, although the parameters used in the present study were different from those used in previous studies.^9^Belal and Yassin^12^ (2014) evaluated the effects of an Er:YAG laser on DT occlusion using SEM to observe melted areas around exposed DTs. The percentage of occluded tubules was found to be significantly greater in the Er:YAG laser group than in the other groups. Moreover, Badran et al.^22^ (2011) reported that 120 s of Er:YAG laser irradiation could lead to complete DT occlusion, showing a wrinkled, melted dentin surface with no visible signs of DTs. In a study of Belal and Yassin^12^ (2014), the laser power (40 mJ, 10 Hz) is slightly lower than that in this study, while the irradiation distance is shorter (the study of Belal and Yassin^12^): slight contact; this study: 30mm). Similarly, the parameter setting in a study by Badran et al.^22^ (2011) is 60 mJ, 2 Hz, (0.12 W), significantly lower than 0.5 W used in this study, but the irradiation time (60 s) is twice the time of 30 s, and there is no water irrigation, which clearly enhances the melting effect of the Er:YAG laser. Overall, the thermomechanical ablation of Er:YAG laser may be a major influencing factor for controlling application parameters of the laser. Temperature increase on the irradiated surface can induce melt and recrystallization of the dentin tissue, resulting in obliteration of the tubule orifices.^8^

Interestingly, we found that the tubule exposure rate increased as the power setting of the laser device increased. In comparison with the dentin surface treated at 0.5 W, 167 J/cm^2^, treatment at 1 W, 334 J/cm^2^ exhibited melting with a bubble-like appearance and a few partially occluded DTs. Our findings were in accordance with those of another study,^23^and this phenomenon can be attributed to the fact that higher power settings may result in rapid water evaporation instead of DT occlusion; the rapid water evaporation results in microexplosions on the irradiated surface, which cause such morphological alterations.^24^ Further, dentin treated at 2 W and 668 J/cm^2^ and dentin treated at 4 W and 1336 J/cm^2^exhibited a similar appearance with a significant difference in the tubule exposure rate (*P* <0.05). We speculated that the similar stripped surfaces were caused by the cutting of superficial hard tissues when the laser power exceeded the ablation threshold. Other studies also showed similar results. Harashima, et al.^25^ (2005) compared morphological features between cavities prepared by an Er:YAG laser and those prepared by an Er,Cr:YSGG laser and found similar, irregular, rugged surfaces with open DTs in both groups. At a wavelength of 2940 nm, the energy of Er:YAG lasers is more strongly absorbed by water than by hard tissues,^26,27^ resulting in microexpansion that can produce hydrokinetic forces for clear and quick removal of the target hard tissue via mechanical separation.^28^

Therefore, based on the SEM images, the parameters 0.5 W and 167 J/cm^2^ seem to be suitable for adequate DT occlusion. Nevertheless, energy accumulation from laser treatment may cause damage to the pulp tissue health. Studies have shown that the pulp would respond to externally applied heat.^29,30^ An intrapulpal temperature increase of 5.5°C could result in necrosis of 15% dental pulp, whereas when the temperature increased by 11°C, pulpal necrosis could occur in 60% of the pulp.

In the present study, Er:YAG laser irradiation at 0.5 W and 167 J/cm^2^ under water and air cooling initially induced a decrease in the intrapulpal temperature (−2.275°C±0.597°C). Similarly, Yaneva et al.^31^ (2016) investigated temperature changes in the pulp chamber during root planing using the Er:YAG laser and found temperature decreases of 1.6°C, 2.4°C, 2.5°C, and 2.5°C after every 10 s. Intrapulpal temperature changes depend on the following factors: the laser emission technique (pulsed or continuous), distance between the applicator and target tissue, wavelength of the laser beam, use of air or water cooling during irradiation, duration of irradiation, and movement of the handpiece.^31^ Thanks to the wavelength, Er:YAG lasers are characterized by a high absorption coefficient, which indicates shallow tissue penetration for both hard and soft tissues.^32^ Therefore, Er:YAG lasers are unlikely to cause adverse thermal effects in tissues. Moreover, pulsed emission of the laser beam can, to some extent, allow for the normalization of the temperature of the irradiated tissue before irradiation by the next laser beam. At the same time, the importance of continuous water and air cooling during irradiation, which prevents an obvious increase in the intrapulpal temperature physically, should not be neglected. Collectively, all the factors described above contributed to the intrapulpal temperature decrease in the air and water cooling group. However, the temperature gradually increased over time, with a change of −1.725°C±0.359°C recorded at 190 s. This indicates that the duration of irradiation is also an important factor for pulp safety. In a previous study, the intrapulpal temperature change within 30 s of Er:YAG laser irradiation under air and water cooling was recorded as −2.2°C±1.5°C. Moreover, intrapulpal temperature gradually increased with longer duration of irradiation, which corroborates the findings of the present study.^33^ We found that the intrapulpal temperature increase during irradiation without water and air cooling was 1.833°C±0.473°C at 30 s and 5.067°C±0.058°C at 60 s, and the final increase was lower than the safe threshold of 5.6°C reported by Zach and Cohen^30^ (1965). Collectively, although water and air cooling during laser irradiation has been demonstrated to be important for pulp safety, the parameters in this study (0.5 W, 167 J/cm^2^) enjoy a highly safety even without cooling.

As for morphological alterations of the pulp tissue, no significant morphological alteration of the odontoblasts was found after treatment with 0.5 W (167 J/cm^2^), according to HE staining. Thus, parametersof 0.5 W (167 J/cm^2^) could be safe for Er:YAG laser treatment for DH.

In summary, we conducted a preliminary *in vitro* study investigating suitable parameters for the successful treatment of DH using the Er:YAG laser. Our findings can, to some extent, serve as a reference for further clinical trials. Taking the high water absorption of Er:YAG laser energy into account, the fluid in teeth and the blood circulation in the pulp may reduce the increase in temperature, consequently increasing the safety of parameters in actual clinical trials. However, this study has several limitations: first, the sample size was relatively small. Second, it is an *in vitro* study, and hence clinical trials with long-term follow-up examinations under intraoral conditions like brushing and acidic challenges are required. Third, it was very difficult to standardize the variations of the DT numbers of dentin even at same depth bellow the dentin due to individual variations. In addition, the pulpal response to this treatment also requires in-depth investigations to further verify its practical safety.

## Conclusions

Our findings suggest that Er:YAG laser irradiation at 0.5 W and 167 J/cm^2^under a water spray at level 1 can effectively occlude DTs without any adverse thermal effects on the pulp.
